# Psychometric properties of the Social Support Scale (SSS) in two Aboriginal samples

**DOI:** 10.1371/journal.pone.0279954

**Published:** 2023-01-03

**Authors:** Pedro Henrique Ribeiro Santiago, Lisa Gaye Smithers, Rachel Roberts, Lisa Jamieson

**Affiliations:** 1 Australian Research Centre for Population Oral Health (ARCPOH), Adelaide Dental School, The University of Adelaide, Adelaide, Australia; 2 School of Public Health, The University of Adelaide, Adelaide, Australia; 3 University of Wollongong, School of Health and Society, Wollongong, Australia; 4 School of Psychology, The University of Adelaide, Adelaide, Australia; Albanian University, ALBANIA

## Abstract

In Australia, despite social support increasingly being reported as playing an important role in influencing health outcomes of Aboriginal and Torres Strait Islanders, measures of social support have not yet been validated for Aboriginal people. The current study aimed to evaluate the validity and reliability of the Social Support Scale in an Aboriginal and/or Torres Strait Islander population. The Social Support Scale (SSS) is a 4-item psychological instrument that was designed to evaluate four social support functions, instrumental, informational, emotional and appraisal support. Data included participants from two different samples: (1) Teeth Talk Study (n = 317), an oral-health randomized controlled trial (RCT) conducted with Aboriginal adults; and (2) the South Australian Aboriginal Birth Cohort Study (n = 367), a prospective longitudinal birth cohort study in which pregnant Aboriginal women were interviewed at baseline. The SSS psychometric properties were examined with Graphical Loglinear Rasch Models (GLLRM). The overall fit to a GLLRM was established (χ^2^(96)_sample1_ = 52.7, p = 0.06; χ^2^(25)_sample2_ = 22.2, p = 0.62) after accounting for local dependence between items 3 and 4. Item 2 displayed differential item functioning by employment status in Sample 1. Regarding dimensionality, the SSS was unidimensional in both samples (γ_obs1_ = 0.80; γ_exp1_ = 0.78, p = 0.65; γ_obs2_ = 0.75, γ_exp2_ = 0.77, p = 0.16). The instrument also displayed good reliability (R_sample1_ = 0.82, R_sample2_ = 0.84). Despite a few identified limitations (such as poor targeting), the findings indicated that the SSS is a promising instrument to provide culturally-valid and reliable measurement of social support among Aboriginal and/or Torres Strait Islander adults. Future studies should further investigate the instrument psychometric properties in other Aboriginal samples and the development and inclusion of culturally-sensitive items are also recommended.

## Introduction

In contemporary Australia, despite improvements due to governmental, non-governmental and private organisations’ efforts over the past decades, the health inequalities between Aboriginal and Torres Strait Islanders and non-Aboriginal Australians persist. For example, Aboriginal people have approximately a 10 years shorter life expectancy and are almost 2 times more likely to commit suicide than non-Aboriginal Australians. A large proportion of this gap can be explained by social determinants of health (e.g. education, employment, discrimination) (31%), while behavioural and biomedical factors (e.g. substance use, physical activity, blood pressure) account for a smaller fraction (11%) AIHW [1]. Among the social determinants of health, social support has been recently receiving attention in Indigenous research.

The effects of social support on health have been extensively reported in non-Indigenous populations. High social support is associated with better outcomes in coronary heart disease [2], type 2 diabetes [3], among many others. In recent years, studies have also investigated the effects of social support on Indigenous health. For example, in Canada social support was consistently associated with better self-reported general health in First Nations, Métis and Inuit [4], while *Yuuyaraq*, social support from extended family and peers, reduced drinking alcohol as a coping response to trauma in Alaska Natives [5].

The research of social support in Aboriginal Australians, however, has been faced with conceptual challenges: the Aboriginal experiences of social and emotional well-being (SEWB) are connected to the well-being of the land and the community; and, for this reason, are not directly comparable to more individualistic western conceptualizations of “*mental* health” [6]. For this reason, to avoid cultural bias, the recommendation is that western-developed psychological instruments should always be validated in an Aboriginal population prior to usage in health research [7].

One western-developed psychological instrument that potentially could be used in Aboriginal research is the Social Support Scale (SSS) (S1 Table) [8]. The SSS was chosen to evaluate social support among Aboriginal Australians for several reasons: (1) despite being brief (and, consequently, reducing response burden), the SSS items measure the four *functions* of social support as contextualised by House (1981) (informational, instrumental, appraisal, and emotional support); (2) these four functions were identified as relevant to understand social support among Indigenous groups (e.g. Canadian First Nations) [9,10]; and (3) the SSS was previously applied among First Nations in Canada (although validation was not conducted) [11]. In general, social support is a broad construct and different (unidimensional or multidimensional) instruments measure distinct aspects of social support, such as sources of social support [12], availability of social support [13], social support functions [8], among others. While there is no single instrument that can cover all aspects of social support [14], the SSS was chosen since the instrument can evaluate the *functions* of social support among Aboriginal Australians. For instance, in case of validity being established, the SSS can indicate whether Aboriginal Australians received instrumental support or emotional support, whereas other instruments (such as the Multidimensional Scale of Perceived Social Support) only assess the sources of social support (e.g. support from family, friends, and significant others) [12]. Furthermore, following the recommendations for cultural adaptation of psychological instruments, the SSS was examined by a 15-member Aboriginal Reference Group, composed of Aboriginal community members and Infant Care workers, which indicated the instrument had content and face validity for Aboriginal and/or Torres Strait Islanders. The advice from the Aboriginal Reference Group further warranted the investigation of the SSS as a potential culturally appropriate instrument for Aboriginal Australians.

### Psychometric properties of the Social Support Scale

The SSS was originally developed by Peeters et al. [8] to evaluate *emotional* (“There are people in my life who pay attention to my feelings and problems”), *appraisal* (“There are people in my life who appreciate what I do”), *instrumental* (“There are people in my life who I can get help from if I need it”) and *informational support* (“There are people in my life who I can talk to about how to handle things”). Additionally, Peeters et al. [8] included two items (“we had a casual chat” and “we made jokes and had fun”) to evaluate *rewarding companionship*. The two items included in the SSS to evaluate reward companionship were not incorporated in the current study since several authors have argued about the importance of “making a clear distinction between supportive interactions and rewarding companionship” [15]. The SSS was composed of 4 items responded according to 5 response categories (0 = Strongly disagree; 1 = Disagree; 2 = Neutral; 3 = Agree; 4 = Strongly Agree). Total scores range from 0 to 16 and higher scores indicate higher social support.

In the original validation of the SSS, the questionnaire was applied to a sample of 41 female secretaries in the Netherlands. A Principal Component Analysis (PCA) was conducted and three components emerged. The first contained the *instrumental* and *informational support* items and was interpreted as “Instrumental Support”, while the second contained the *emotional* and *appraisal support* items and was interpreted as “Intimate Support”. The third component included the two *rewarding companionship* items. The study also evaluated the reliability (α_*Instrumental*_ = 0.80; α_*Intimate*_ = 0.77; α_*Rewarding*_ = 0.76) and the correlation between subscales, such as the correlation between the Instrumental and the Intimate Support subscales (r = 0.56; p < 0.001) [8].

Despite reporting adequate psychometric properties for a limited western population of female secretaries, the restricted sample of the original study, combined with the cultural differences of Aboriginal Australians, limits the generalizability of the results (number of dimensions, magnitude of the loadings) to an Aboriginal population. After the initial validation conducted by Peeters et al. [8], the SSS was applied in epidemiological studies but no further validation has been conducted.

### The present research

Research on social support can provide insight into the social determinants that can contribute to the existing health gap between Aboriginal and non-Aboriginal Australians. To the best of our knowledge, there are no available validated instruments to measure social support in any Indigenous population. Therefore, the aim of the current study was to evaluate whether the SSS is a valid and reliable measure of social support in Aboriginal Australians.

## Methods

### Participants and procedures

*Sample 1*: The sample included 317 Aboriginal Australians that participated in the Teeth Talk Study (TT), a randomized controlled trial (RCT) intended to improve oral health literacy among Aboriginal adults in Port Augusta. Ethical approval was received from the Aboriginal Health Council of South Australia and the Human Research Ethics Committee of the University of Adelaide. Ethical approval was also given by the Board of Management of the Pika Wiya Health Service (PWHS), the local community controlled Indigenous health service. The study was publicised through posters in community centres, in addition to advertisements on an Aboriginal radio station. The recruitment included a variety of methods, such as self-nomination, word of mouth, home visits and referrals. For more information on the TT study, please refer to Parker, Misan [16].

*Sample 2*: The sample was composed of 367 pregnant Aboriginal women interviewed at the baseline of the South Australian Aboriginal Birth Cohort (SAABC). SAABC is a prospective longitudinal birth cohort study initiated as a randomized controlled trial aimed at reducing early childhood caries of Aboriginal children in South Australia. The SAABC includes 2-, 3-, 5- and 7-year-old follow-ups and the families and children are currently participating in the 9-year-old follow-up. The study received approval from the University of Adelaide Human Research Ethics Committee, the Aboriginal Health Council of South Australia, the Government of South Australia and the Human Research Ethics Committees of three participating South Australian hospitals [17]. For more information on the SAABC, please see Jamieson, Hedges [18]. The psychological measures (including the SSS) were included in a broader questionnaire administered to the study participants at baseline.

Written informed consent was obtained from all individual participants included in both studies. Additionally, all procedures performed in the TT and SAABC studies were in accordance with the ethical standards of the institutional and/or national research committee and with the 1964 Helsinki declaration and its later amendments or comparable ethical standards.

### Complementary measures

*The Perceived Stress Scale (PSS)*: The PSS is the most widely used psychological instrument to measure perceived stress, which evaluates if a person’s life is perceived as unpredictable, uncontrollable, or overloading. The PSS is composed of 14 items in its original form (PSS-14) and the subscales of Perceived Stress (PS) and Perceived Coping (PC). A revised version with 13 items was validated in an Aboriginal population [7]. The PSS was responded according to 5 response categories (0 = Not at all; 1 = Rarely; 2 = Sometimes; 3 = Fairly often; 4 = Very often). For the PS subscale, total subscale scores ranged from 0 to 28 and higher scores indicate higher perceived stress. For the PC subscale, total subscale scores ranged from 0 to 24 and higher scores indicate higher perceived coping.

### The Rasch measurement models

The Rasch model (RM) is part of the family of Item Response Theory (IRT) models and its development aimed to provide scientific measurement in social sciences according to the fundamental measurement properties such as *objectivity* [19]. In natural sciences (such as physics or chemistry), the measurement of features that can be directly observed (e.g. height) or cannot be directly observed (e.g. temperature) is objective since it is independent of the measurement instrument being used (e.g. ruler or thermometer) [20]. For example, the measured temperature of an individual is independent of the thermometer being used and different thermometers should indicate the same temperature (considering small variations due to random measurement error) [21]. Similarly, Rasch [22] argued that measurement in social sciences is objective once “comparisons between individuals become independent of which particular instruments–tests, or items or other stimuli–within the class considered have been used”. The provision of objective measurement (specific within a frame of reference) is one feature of the RM that is distinctive from other IRT models [19]. Another distinctive feature of the RM is that the sum score is a sufficient statistic for the person parameter. This indicates that the instrument sum score, which is calculated by summing all individual items, includes all the information available about the latent trait and no additional information would be added by knowing individual item scores [19]. Furthermore, items fitting a Rasch model exhibit the following measurement properties: (1) unidimensionality: the items measure a unique latent trait (i.e. social support); (2) homogeneity: the order of item difficulty (i.e. more difficult to less difficult item) is equal for all respondents; (3) monotonicity: the endorsement of “higher” categories is an increasing function of the latent trait; (4) local independence: items are conditionally independent given the latent trait; and (5) absence of differential item functioning: items are conditionally independent of exogenous variables given the latent trait [20,23].

As abovementioned, two main requirements for items fitting a RM are local independence and the absence of differential item functioning (DIF). Regarding local independence, psychological instruments are developed to measure a latent trait (e.g. an unobservable psychosocial process such as social support) and the latent trait should be the common cause generating the item responses. *Ideally*, in a psychological instrument, the item responses should be influenced only by the latent trait and nothing else. The influence of the latent trait is what makes the item responses (marginally) correlated in the data. Using the SSS instrumental and informational support items as an example, it is expected that participants who report high instrumental support (item 3) *on average* will also report high informational support (item 4). This occurs since participants with high social support (i.e. latent trait) will receive multiple forms of social support (e.g. instrumental, informational support) in their lives, so scores from these two items will be positively correlated. The requirement of *local independence* is that items should not be correlated anymore once the influence of the latent trait is accounted for. That is, for respondents with the *same level of the latent trait* (i.e. same level of social support), there should no correlation between the informational and instrumental support items. In case the correlation still exists (after the influence of the latent trait is accounted for), it indicates that the items are correlated for other (undesirable) reasons beyond the influence of the latent trait, such as redundancy between items or items that are too similar in content [24]. When the items are not independent given the latent trait, this is referred to as *local dependence* (LD).

Similarly, items responses are influenced by respondents’ characteristics through the latent trait. For example, it is expected that respondents with higher educational attainment (i.e. sociodemographic characteristic) will experience higher social support (i.e. latent trait) and, consequently, will have higher scores on the SSS items (e.g. higher scores on the instrumental support item). Hence, the item responses will be (marginally) correlated with respondents’ characteristics (e.g. scores on the instrumental support item will be positively correlated with educational attainment). However, once the influence of the latent trait is accounted for, there should be no more correlation between the item responses and respondents’ characteristics. For example, for respondents with the *same level of the latent trait* (i.e. same level of social support), there should no correlation between the informational support item and educational attainment. In case the correlation still exists (after the influence of the latent trait is accounted for), it indicates that the respondent characteristics is influencing the item responses for other (undesirable) reasons beyond the influence of the latent trait. For instance, respondents with low educational attainment may have been unable to understand the item, influencing their item responses but without any relation to their level of social support. In this case, when an item is not independent of respondents’ characteristics given the latent trait, this is referred to as *differential item functioning* (DIF).

In summary, the RM implies that items are conditionally independent to other items (i.e. local independence) and exogenous variables (i.e. absence of differential item functioning (DIF)) given the latent trait. However, these mathematical requirements of the model are strict, and it is common for items of rating scales applied in health sciences to exhibit LD and/or DIF. In this case, items with LD and/or DIF would not fit a RM and would need to be removed from the questionnaire for the remaining items to obtain fit to the RM. However, this practice is problematic since the removed items can be relevant in terms of their content and they might cover important aspects of the construct being measured [25]. For this reason, models called Graphical Loglinear Rasch Model (GLLRM) which extend the RM with log-linear parameters were developed to incorporate items with uniform LD and uniform DIF (i.e. the term uniform is omitted from now on in the manuscript when referring to uniform LD or uniform DIF). Considering that the RM (such as the Partial Credit Model) does not enable items with LD and/or DIF, GLLRMs extend the RM to include new parameters that can model items with LD and/or DIF. Hence, GLLRMs enable items with LD and/or DIF to be retained in the questionnaire while still providing (essentially) objective measurement, which is a distinctive feature of the RM. For an in-depth discussion about objective measurement and (essentially) objective measurement, please refer to Kreiner [19] and Kreiner and Christensen [26]. In practice, when items with LD and/or DIF are retained in the questionnaire, items with LD convey less information than independent items and items with DIF require adjustment of scores between subgroups. However, in both cases, the items serve the purpose of measuring the latent trait and retaining them contribute to construct validity [27].

### Statistical analysis

*Item analysis*: Initially, the fit of the items to the RM was tested. In case there was no fit, it was investigated whether the departures in terms of positive LD and/or DIF could be accounted by a GLLRM. All statistical analyses were conducted with the DIGRAM v4.05 [28]. Considering that missing values for individual items ranged from 0.0% to 0.005% considering both studies, multiple imputation was not required [29].

*Model fit and item fit*: Overall fit of the model was evaluated through the Conditional Likelihood Ratio (CLR) test [30]. The CLR test evaluates *measurement invariance* within subgroups, providing a test of overall model fit (i.e. subgroups defined by higher and lower scores) and overall DIF (e.g. subgroups defined by exogenous variables, such as males and females). In the current study, the characteristics analysed for DIF were sex (Male; Female), education (education level up to High School; TAFE or University) and employment status (Not employed; Employed). Technical and Further Education (or TAFE) is the biggest provider of post-secondary education in Australia. Item fit was evaluated by conditional infit and outfit statistics [31,32].

*LD and DIF*: LD was evaluated through the matrix of residual correlations. Since residuals correlations of the Rasch model are known to be *negatively biased*, the mean-adjusted residual correlation matrix was used ($Q_{3}^{*}$ statistic) [24]. In addition, Kelderman’s [33] likelihood ratio (LR) test was conducted to evaluate LD and/or DIF, and the *magnitude* of the LD/DIF was informed through partial Goodman-Kruskal *γ* rank correlations. Partial Goodman-Kruskal *γ* rank correlations were classified as weak (< 0.15), moderate (0.15–0.30), or strong (> 0.30) [34]. To evaluate LD, Kelderman’s LR test compared the model after the inclusion of a uniform LD parameter with the fitted model (without the inclusion of the uniform LD parameter) for each possible item pair (e.g. LD between Item 1 and Item 2, LD between Item 1 and Item 3, etc). To evaluate DIF, Kelderman’s LR test compared the model after the inclusion of a uniform DIF parameter with the fitted model (without the inclusion of the uniform DIF parameter) for each possible item covariate pair (e.g. DIF between Item 1 and Education, DIF between Item 2 and Education, etc). Due to multiple testing, the Benjamini–Hochberg procedure was used to adjust the *p*-values to control the false discovery rate (FDR) [35]. Significance was evaluated at a 5% critical level (after adjusting *p*-values to control the FDR). In case DIF was found, we report conversion tables for score adjustment between groups.

*Dimensionality*: The SSS was divided into two subscales composed of Items 1 and 2 (“Emotional Support”), and Items 3 and 4 (“Instrumental Support”), and a formal test of dimensionality was conducted by comparing the observed *γ* correlation of the subscales with the expected *γ* correlation of the subscales under an *unidimensional* model. The rationale of this analysis was that the correlation between two subscales measuring distinct traits (i.e. “Emotional Support” and “Instrumental Support”) should be weaker than the expected correlation of subscales measuring the same latent trait (i.e. “Social Support”). The division of the SSS into two subscales (“Emotional Support” and “Instrumental Support”) was made based on theoretical and empirical considerations since these two subscales were identified as meaningful dimensions of social support in the original validation of the SSS (considering also evidence from previous research) [8]. However, it is still required to investigate whether the two dimensions can be empirically identified in the item responses from participants of the SAABC and TT studies. Furthermore, other researchers such as Semmer et al. [36] have argued that the distinction between “Instrumental Support” and “Emotional Support” is tenuous since skilful social support usually simultaneously provides instrumental support (i.e. by helping those receiving it deal with practical problems) and emotional support (i.e. by communicating empathy, caring and respect to those receiving it). As such, these two aspects of social support (“Instrumental and Emotional”) can potentially be better understood as a unique dimension of social support. The unidimensionality (in comparison to multidimensionality) of social support has been discussed in the literature [37].

*Reliability and targeting*: Since Cronbach’s *α* provides a lower-bound estimate of reliability *when items are locally independent*, a Monte Carlo simulation method [38] that accounts for LD between items was applied. Targeting refers to whether the levels of the latent trait evaluated by the items match with the levels of the latent trait observed in the population. For example, whether the levels of social support measured by the SSS items match with the levels of social support observed in Aboriginal populations. The targeting was evaluated: (1) statistically through the Test Target Information Index, which is the mean test information divided by the maximum obtained test information [39]; and (2) graphically through the item map, by comparing the distribution of person parameters location to the distribution of item thresholds location. Furthermore, we also evaluated measurement precision with a graph indicating the test information and the Standard Error of Measurement (SEM) across the latent trait.

*Criterion validity*: Considering that scores are ordinal, the non-parametric Kendall’s τ correlation of the SSS with the PSS was calculated. It was expected a positive correlation of the SSS with PC subscale and a negative correlation with the PS subscale. The Kendall’s τ was chosen for the analysis of convergent validity since Pearson’s *r* product-moment correlation requires *continuous* and *normally* distributed variables and the SSS item responses are *discrete* and *ordinal*. In these cases, the Kendall’s τ should be employed and the correlation coefficient will be calculated from the observations’ ranks rather than their actual values [40]. Previous meta-analyses have indicated negative small effect size Pearson correlations between social support and stress (ranging from -0.14 to -0.19) and positive medium effect size Pearson correlations between social support and coping (ranging from 0.41 to 0.51) [41].

## Results

The sample characteristics of both studies are found in Table 1.

**Table 1:** Characteristic of the study participants.

	Sample 1		Sample 2	
	n	%	n	%
Age				
Mean	36.4		24.9	
SD	14.0		5.9	
Min/Max	18/82		14/43	
Missing	0	0%	0	0%
Sex				
Female	214	76%	367	100%
Male	103	24%	0	0%
Missing	0	0%	0	0%
Education				
High school or less	236	74%	266	73%
TAFE or university	81	26%	98	27%
Missing	0	0%	0	0%
Employment status				
Employed	80	25%	45	12.4%
Not employed	237	75%	316	87.3%
Missing	0	0%	1	0.3%

Note. Mean values, range, and standard deviations; numbers and percentages.

TAFE, Technical and Further Education (trade school/college).

*Dimensionality*: The correlation between subset 1 (Item 1 and Item 2) and subset 2 (Item 3 and Item 4) was in accordance with the expected correlation under a unidimensional model in Sample 1 (γ_obs_ = 0.796, γ_exp_ = 0.781, p = 0.654) and Sample 2 (γ_obs_ = 0.747, γ_exp_ = 0.768, p = 0.163). Therefore, the results indicated that the SSS is a unidimensional scale and the four items were evaluated together.

*Sample 1*: The CLR indicated that item parameters were not invariant between participants with low scores and high scores (χ^2^ = 69.6; df = 15; p<0.001) and, therefore, there was no overall fit to the RM. When item parameters were compared across subgroups, invariance was not achieved between employed and not employed (χ^2^ = 31.2; df = 15; p = 0.008) indicating DIF by employment status (Table 2).

**Table 2:** Conditional likelihood ratio test of overall model fit and global DIF.

	Sample 1				Sample 2		
Model	Homogeneity	DIF by sex	DIF by education	DIF by employment status	Homogeneity	DIF by education	DIF by employment status
RM	χ^2^(15) = 69.6, p<0.001	χ^2^(15) = 15.8, p = 0.392	χ^2^(15) = 13.6, p = 0.554	χ^2^(15) = 31.2, p = 0.008	χ^2^(15) = 25.1, p = 0.049	χ^2^(15) = 19.3, p = 0.200	χ^2^(15) = 12.8, p = 0.621
GLLRM	χ^2^(38) = 52.7, p = 0.057	χ^2^(38) = 50.4, p = 0.086	χ^2^(38) = 39.5, p = 0.397	χ^2^(30) = 43.9, p = 0.048	χ^2^(25) = 22.2, p = 0.625	χ^2^(25) = 10.3, p = 0.996	χ^2^(25) = 16.4, p = 0.903

Note. The subgroups were defined according to lower and higher scores (i.e. homogeneity) to evaluate overall model fit; and according to sex (Men; Women), education (Up to high school; TAFE or University) and employment status (Not employed; Employed) to evaluate overall DIF.

The conditional outfits and infits disclosed misfit of Item 1 (Outfit = 1.350, SE = 0.103, p<0.001; Infit = 1.319, SE = 0.100, p = 0.001) (S2 Table).

Before considering removing Item 1, it was investigated whether the departures from the RM would constitute LD and/or DIF. The analysis of the residual correlations (S3 Table) suggested that, after the influence of the trait (“social support”) was accounted, there was LD between Item 3 (“There are people in my life who I can get help from if I need it”) and Item 4 (“There are people in my life who I can talk to about how to handle things”) ($Q_{3}^{*}$ = 0.221; p<0.001). The Kelderman’s LR test provided evidence of LD between items 3 and 4 (LR = 57.26, df = 16, p<0.001) (γ_avg_ = 0.50) and items 1 and 2 (LR = 68.46, df = 16, p<0.001; γ_avg_ = 0.02). However, in the last case, the magnitude of the dependence (γ_avg_ = 0.02) was unsubstantial. Furthermore, the results from Kelderman’s LR tests disclosed that Item 2 had exhibited moderate DIF by employment status (LR = 12.60, df = 4, p = 0.013; γ = 0.21). The DIF indicates that SSS scores cannot be compared between not employed and employed respondents without adjustment since differences between groups in their (not adjusted) total scores will not reflect *true* differences in their level of social support. Hence, to enable the comparison between groups, we present a conversion table for score adjustment between respondents that were not employed and employed (Table 3). The *adjusted* scores can be used to provide a direct comparison between not employed and employed respondents.

**Table 3:** Conversion table for score adjustment.

Scores	Adjusted Scores	
Social Support Scale	Employment status: Not employed	Employment status: Employed
1	1.00	1.72
2	2.00	3.32
3	3.00	4.64
4	4.00	5.79
5	5.00	6.74
6	6.00	7.53
7	7.00	8.20
8	8.00	8.82
9	9.00	9.45
10	10.00	10.13
11	11.00	10.92
12	12.00	11.88
13	13.00	12.96
14	14.00	14.03
15	15.00	15.05

Note. The table indicates conversion values for comparison among subgroups. For example, if an Aboriginal respondent not employed had on the SSS an observed total score of 10, the score should be adjusted to the new (adjusted) total score of 10.13. Values for extreme total scores (0 and 16) are not provided.

The impact of the moderate DIF by employment status can be observed in the differences between the scores and the adjusted scores. For instance, the differences between the scores and adjusted scores were especially high among those with lower social support (e.g. a score of 1.00 among those not employed would correspond to a score of 1.72 among those employed) compared to those with higher social support (e.g. a score of 15.00 among those not employed would correspond to a score of 15.05 among those employed).

**Fig 1:**
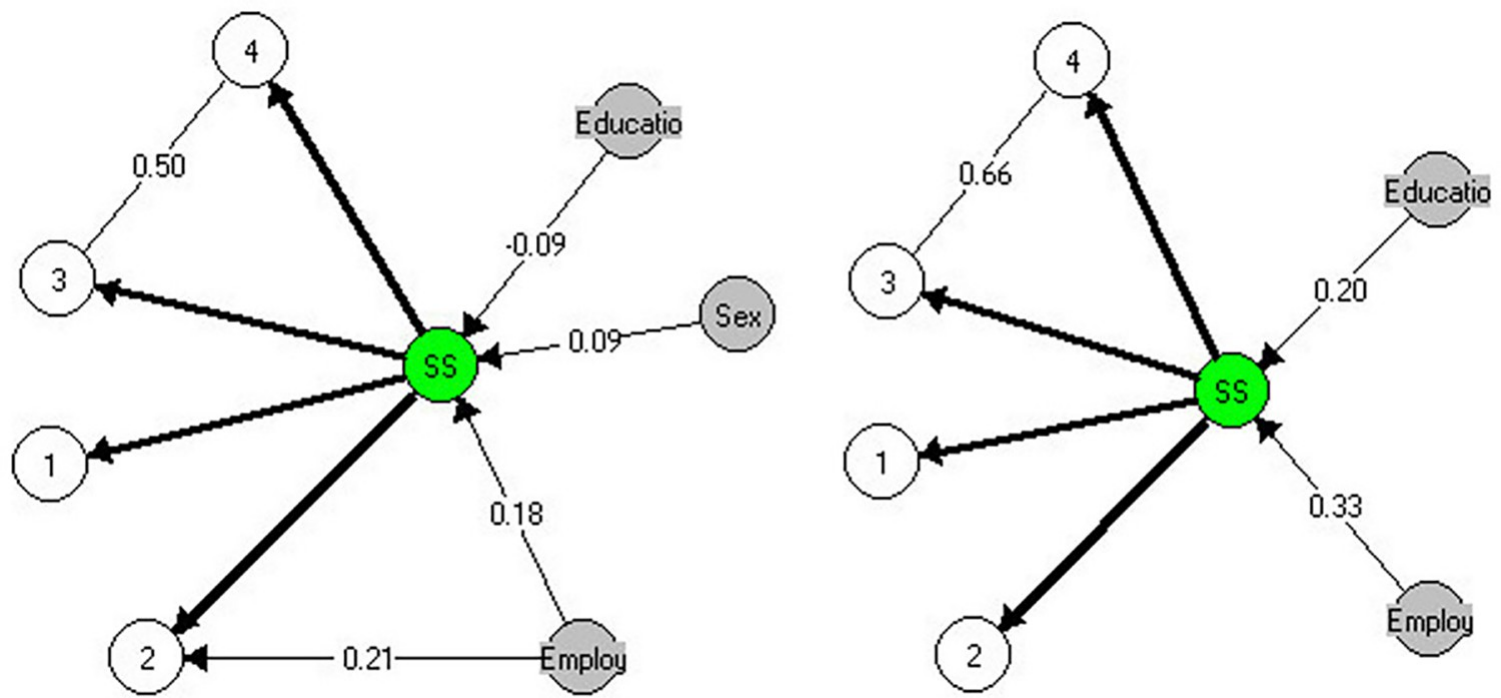
GLLRMs of the SSS for sample 1 (left) and sample 2 (right). Note. The Markov graph nodes represent the item numbers, the exogenous variables, and the latent trait. Disconnected nodes indicate that variables are conditionally independent and partial *γ* inform the magnitude of the LD and DIF. When LD/DIF was unsubstantial (*γ*<0.1), the edges were omitted (see S1 Fig).

Fit to a GLLRM adjusting for these departures was found (χ^2^ = 52.7, df = 96, p = 0.057) (Table 2) (Fig 1).

**Fig 2:**
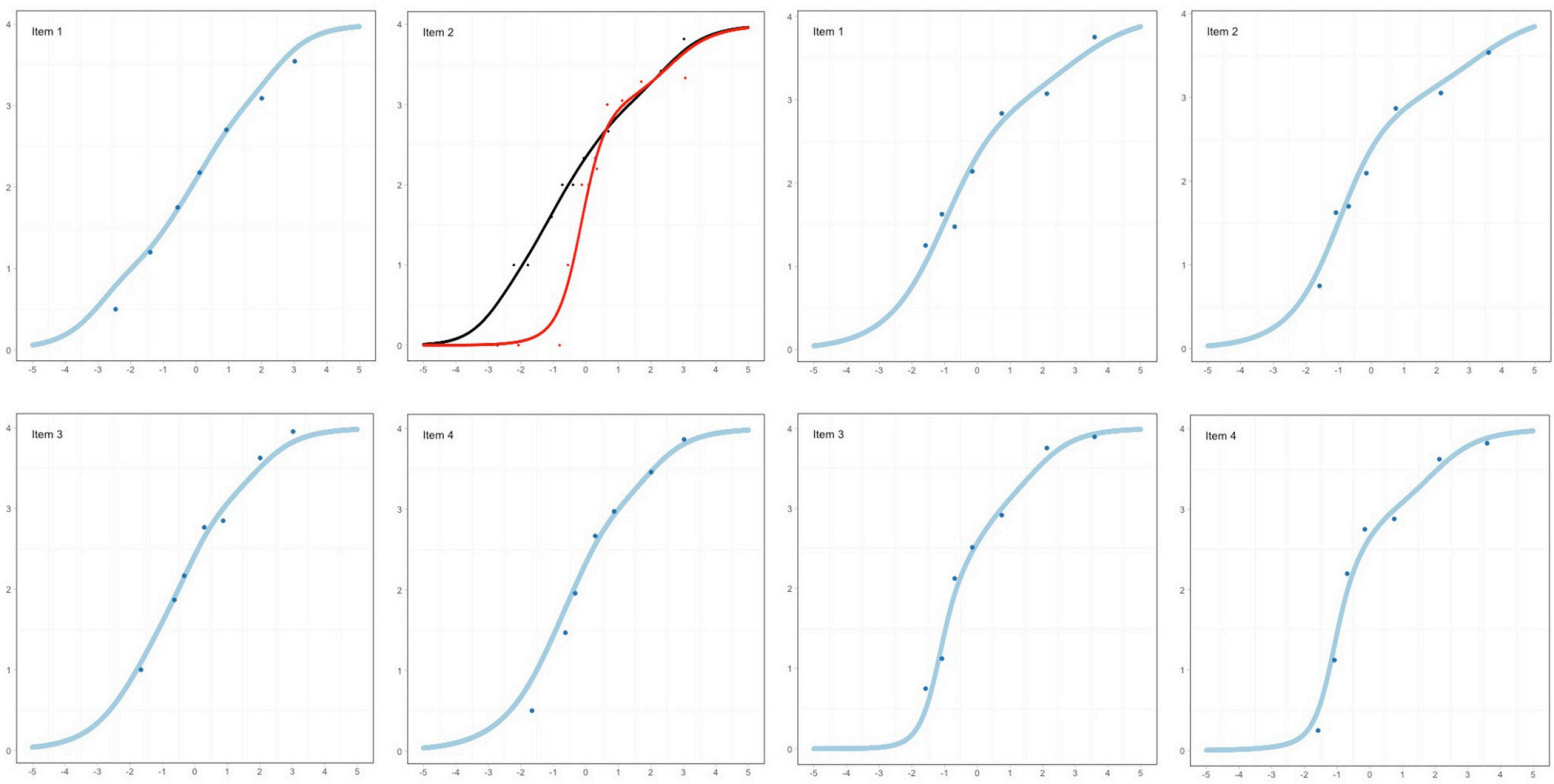
Item characteristic curves of the GLLRM for sample 1 (left) and sample 2 (right). Note. The x-axis indicates the latent trait (“Social support”) and the y-axis indicates the item score. The points represent the *average* observed item responses in each of the seven class intervals. Since Item 2 had DIF by employment status, the Item Characteristic Curves (ICCs) for participants employed (black) and not employed (red) are displayed.

Considering that there was no further evidence of item misfit (S2 Table) (Fig 2), and the Kelderman’s LR indicated no substantial evidence of DIF or LD (S4 Table), the measurement model for the SSS in Sample 1 was established.

*Sample 2*: The CLR indicated weak evidence against the RM (χ^2^ = 25.1; df = 15; p = 0.049). Invariance of item parameters was found within subgroups, and there was no evidence of DIF by education (χ^2^ = 19.3; df = 15; p = 0.200) or employment status (χ^2^ = 12.8; df = 15; p = 0.621) (Table 2). In addition, there was no item misfit (S2 Table). However, the examination of the residual correlations showed a similar pattern of Sample 1, suggesting LD between Item 3 and Item 4 ($Q_{3}^{*}$ = 0.316; p<0.001) (S3 Table). The Kelderman’s LR test disclosed LD between items 3 and 4 (LR = 68.15, df = 16, p<0.001; γ_avg_ = 0.66) and items 1 and 2 (LR = 44.33, df = 16, p<0.001; γ _avg_ = 0.15). Finally, Kelderman’s LR test showed no evidence of DIF.

The strong LD between items 3 and 4 was the reason why the CLR initially showed evidence against the RM since after this LD parameter was incorporated into a GLLRM, the model fitted (χ^2^ = 22.2, df = 25, p = 0.625) (Table 2) (Fig 2). Since there was no further evidence of item misfit (S2 Table) and LD or DIF (S4 Table), the measurement model for the SSS in Sample 2 was established.

*Reliability and targeting*: The SSS displayed good overall reliability (R_sample1_ = 0.82, R _sample2_ = 0.84) and adequate overall probability of person separation (P_sample1_ = 0.77, P _sample2_ = 0.78) (S5 Table). Therefore, if we order two randomly chosen participants of Sample 2 based on their total score in 78% of the cases they will be correctly ordered with respect to their *true* level of social support.

Targeting was poor in both samples (S5 Table). For example, the Test Information Target Index showed that in Sample 1 the SSS provided only 28% of the total information available regarding social support in comparison to a perfectly targeted instrument. The reason is that the mean score in Sample 2 was 12.49 (SD = 3.15) while the SSS was perfectly target for a group with a mean score of 7.47 (S2 Fig).

**Fig 3:**
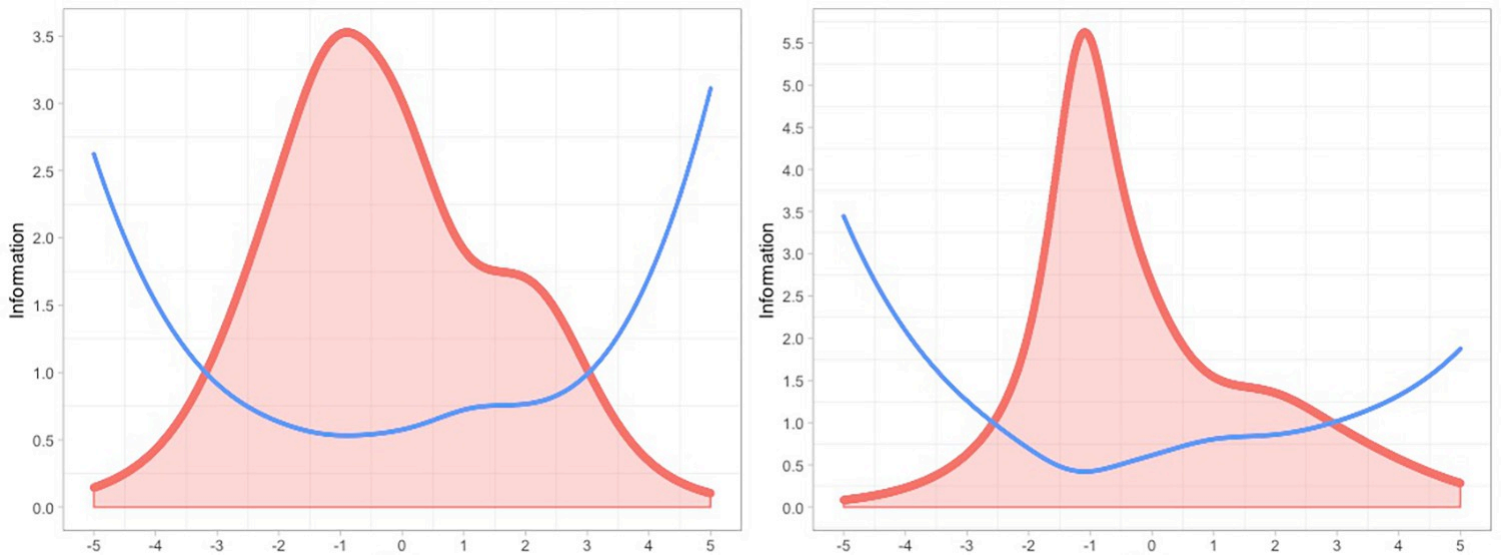
Test Information function for sample 1 (left) and sample 2 (right). Note. The x-axis indicates the latent trait (“Social support”) and the y-axis indicates the information. The red line with shaded area represents the Test Information Function and the blue line represents the Standard Error of Measurement (SEM).

The Test Information Function for both samples is displayed in Fig 3.

Considering that Item 2 had DIF by employment status, we also report the Test Information for Sample 1 among the not employed and the Test Information for Sample 1 among the employed (S3 Fig). The examination of Fig 3 (and also S3 Fig) indicates that, in both Samples 1 and 2, the SEM was lower around the range of -3 to 3 logits, steeply increasing outside of this range. The examination of item maps (S2 Fig) displays how, across both samples, there were a high proportion of respondents with values higher than 3 logits on the latent trait. Hence, the measurement precision of the SSS decreased (i.e. higher SEM) for respondents with high social support (i.e. person parameters higher than 3 logits). These findings further confirm that the SSS was not perfectly targeted for Aboriginal Australians with strong social support and indicate that measurement precision decreased in this group.

*Criterion validity*: The SSS score displayed the expected pattern of convergent and divergent validity regarding perceived stress (r_sample2_ = -0.21, 95% C.I. [-0.35, -0.06]) and perceived coping (r _sample1_ = 0.12, 95% C.I. [0.01, 0.22], r _sample2_ = 0.28, 95% C.I. [0.13, 0.41]) on Sample 2. However, the magnitude of the Kendall’s τ correlation observed on Sample 1 regarding perceived stress (r_sample1_ = -0.04, 95% C.I. [-0.15, 0.07]) was weaker.

One important aspect of the analysis of convergent validity is the interpretation of Kendall’s τ [42] rank correlation coefficients, which ranged from -0.21 (between social support and perceived stress in Sample 2) to 0.28 (between social support and perceived coping in Sample 2). The Kendall’s τ is an *ordinal* correlation coefficient and the usual effect size interpretation of .10 to .30 (small), .30 to .50 (medium) and .50 to 1.00 (large) of Pearson’s product-moment correlations [43] does not apply. The reason is that, despite both statistics ranging from -1 to +1, the values of the Pearson’s correlations are approximately 1.5 times higher than Kendall’s τ making these statistics not directly comparable [44]. The *ordinal* correlations found in our study which ranged from -0.21 to 0.28 would correspond to Pearson’s correlations ranging from -0.32 to 0.43 [44,45]. Therefore, except for the correlation between social support and perceived stress in Sample 1 (r_sample1_ = -0.04, 95% C.I. [-0.15, 0.07]), all correlations found in the analysis of criterion validity had *medium* effect sizes.

## Discussion

The current study aimed to evaluate the validity and reliability of the SSS in an Aboriginal and/or Torres Strait Islander population. The results indicate that the SSS is a measure that is valid and reliable to measure social support in Aboriginal and/or Torres Strait Islander population, and the replication of the findings across samples provides confidence in the robustness of the results.

*Dimensionality and local dependence*: The results showed that the SSS is a unidimensional scale with strong local dependence between Item 3 (“There are people in my life who I can get help from if I need it”) which measures *instrumental support* and Item 4 (“There are people in my life who I can talk to about how to handle things”) which measures *informational support*. The dependence between the instrumental and informational items is in accordance with psychological theory since providing useful information is closely related to directly helping the completion of a task [46].

The unidimensionality of the SSS is consistent with recent research on social support. Semmer, Elfering [36] argued that the distinction between the two dimensions of Emotional and Instrumental support, previously reported in empirical research, is tenuous since many supportive behaviours described as instrumental (e.g. helping to clean the house) have also an emotional meaning by communicating empathy, caring and respect. Hence, Semmer et al. [36] explained that: “Skillfully provided social support will, therefore, often be both instrumental in behavior and emotional in symbolic meaning. At least for support that is interpreted as helpful, a high correlation between instrumental and emotional support from the same source is, therefore, very likely. This might explain the observation that instrumental and emotional support are often strongly correlated in previous research”.

Considering that psychological theory and previous research evidence were informing the SSS dimensionality and potential dependencies between the items (e.g. potential dependence between *instrumental* and *informational support*), the dimensionality analysis and the subsequent establishment of GLLRMs were conducted in our study based on substantive theory. For example, according to psychological theory, the items *instrumental* and *informational support* would represent “Instrumental Support”, while the items *emotional* and *appraisal support* would represent “Intimate Support” [8]. Hence, in the dimensionality analysis conducted in our study, the items *instrumental* and *informational support* were divided into a subset, while the items *emotional* and *appraisal support* were divided into another subset, to evaluate whether these pairs would constitute separate dimensions. Similarly, when we evaluated which additional parameters to include in the GLLRMs, the LD between the items *instrumental* and *informational support* not only was observed in the data (with a strong magnitude) but was also consistent with previous psychological research [46], substantiating our decision to include the LD parameter in both samples. As such, in our study, the GLLRMs were established in an exploratory manner since there were previous psychological theory and evidence that guided the decisions regarding which parameters to include (although other procedures to establish the GLLRMs have been proposed in the literature, such as *item screening* [47]).

*DIF*: Item 2 (“There are people in my life who appreciate what I do”) displayed DIF by employment status. This indicates that employed Aboriginal respondents systematically endorsed that there are people in their life who appreciate what they do in comparison to non-employed respondents given the same level of social support. High unemployment rates are one of the main social inequalities experienced by Aboriginal Australians; for example, 75% of participants in Sample 1 and 87% in Sample 2 were not employed. Considering that the causes of unemployment among Aboriginal Australians are rooted in historical and cultural factors, it seems plausible that individuals who were employed systematically felt more appreciated by those in their life. This is a potential reason for the DIF by employment status observed in Item 2.

Moreover, there is one possible explanation why Item 2 DIF by employment status was not replicated in Sample 2, which comprised pregnant Aboriginal women. In Australia, approximately 23% of the women leave their job during pregnancy and 92% take a paid or unpaid leave [48]. Since it is common to be not employed or absent from work during pregnancy, Aboriginal mothers who were employed during their gestation might not have felt systematically more appreciated by others in comparison to the non-employed mothers. Due to the absence of DIF among Aboriginal pregnant women, the conversion table for score adjustment should not be employed among Aboriginal pregnant women. In summary, we recommend that future studies should try to replicate the results found in Sample 1 and investigate again if Item 2 displays DIF by employment status.

Finally, an additional possibility would be to combine participants from both samples and evaluated DIF according to the sample. For example, include all women from Samples 1 and 2 into a new sample and evaluate DIF according to which sample they originally belonged. We decided, however, to not prioritise this analysis for two reasons. Firstly, considering that we had data available on the SSS from two *independent* studies with Aboriginal Australians (and considering the difficulties in recruiting and retaining Indigenous participants [49,50]), this could provide a unique opportunity to evaluate whether the SSS psychometric properties would replicate across independent samples. For this reason, we decided to maintain the samples separately to check the reproducibility of the findings, which later indicated that the unidimensionality of the SSS and the LD between Items 3 and 4 indeed replicated across samples. Secondly, considering that both samples were very distinct (for instance, all women in Sample 2 were *pregnant* during data collection), we considered it more relevant to evaluate DIF *within each sample first* according to the respondents’ background characteristics. As abovementioned, Item 2 displayed DIF by employment status in Sample 1 but not Sample 2 and the absence of DIF by employment status in Sample 2 was plausibly related to the women’s pregnancy. Since item parameters were already distinct for these two groups in Sample 1 (i.e. employed and not employed), any comparison with females from Sample 2 would require now a comparison between one of the two groups from Sample 1 and the females from Sample 2. However, this analysis and the subsequent interpretation of the findings would be less conceptually clear. In summary, given how distinct the two samples were, we considered it more important to first investigate the instrument functioning within each sample, evaluating and interpreting DIF according to the respondents’ characteristics, before conducting any analysis directly comparing both samples.

*Reliability and targeting*: Reliability and probability of person separation were good in both samples (R_sample1_ = 0.82, R _sample2_ = 0.84, P_sample1_ = 0.77, P _sample2_ = 0.78). Reliability values between .70 and .80 are usually deemed adequate for research purposes, while values between .80 and .85 indicate that the instrument can be used for individual testing in low-stakes scenarios [51].

Targeting was poor in both samples since participants had more social support than the levels the instrument was developed to measure. In Indigenous groups, high levels of social support do not implicate *only* health protection. The high-density social networks (e.g. extended families) provide support and reinforce belonging but also exert conformity pressure and over-obligations. Furthermore, the low income and poor material circumstances confine individuals within their immediate social context, making it harder to avoid harmful relationships (e.g. domestic violence) [10]. To improve targeting, future studies can further develop the SSS by creating *culturally-sensitive difficult* items to measure higher levels of social support. For example, an item could measure whether Aboriginal Australians experience social support in the workplace or from the wider Australian community (i.e. non-Indigenous Australians), and this would be a difficult item that could improve the targeting of the SSS. Future studies should investigate the SSS psychometric properties in other Aboriginal samples, specifically samples with lower levels of social support, to further examine whether the SSS targeting improves.

*Criterion validity*: The analysis indicated a negative association with perceived stress (since Aboriginal Australians with higher social support perceived themselves to be less stressed) and a positive association of social support with perceived control. The only exception was the correlation between social support and perceived stress in Sample 1 which was not meaningful. In general, the patterns of convergent and divergent validity were congruent with the theoretical expectations and previous empirical research. While the effect size of one of the correlations observed in our study (i.e. medium effect size) between social support and stress was slightly higher than effect sizes previously reported (i.e. small effect size), the medium effect sizes observed in our study of the correlations between stress and coping were congruent with the effects previously reported in meta-analyses [41]. Furthermore, in both cases (stress and coping), the direction of the observed correlation was consistent with meta-analytical research (i.e. negative correlations between social support and stress and positive correlations between social support and coping) [41], providing further evidence of the SSS construct validity.

*Strengths and limitations*: The strengths of the present study comprised of the modern psychometric methodology applied and the use of two Aboriginal samples to ensure replicability of the results. One fundamental limitation is that the main sample of this study which contained Aboriginal men and women was a convenience sample in a rural setting; while the second sample was recruited in metropolitan areas but interviewed only pregnant Aboriginal women. Given the low percentage of males in Sample 1 (24%) and the absence of males in Sample 2, DIF by sex needs to be re-evaluated in other samples, to confirm our findings indicating no DIF by sex. Another important characteristic to be evaluated in future studies is DIF by age, considering that participants in Samples 1 and 2 were young (on average around 35 and 25 years, respectively). Overall, future studies are required to investigate the construct validity of the SSS in more representative samples of the Aboriginal population. Another limitation is that only a single measure (the PSS) was included to evaluate criterion validity. Future studies should further evaluate the criterion validity of the SSS by examining its associations with other selected measures. Furthermore, the SSS is a brief unidimensional instrument that only measures the *functions* of social support (informational, instrumental, appraisal, and emotional support). Future studies should also investigate among Aboriginal Australians the psychometric properties of (unidimensional or multidimensional) instruments that measure other aspects of social support, such as sources of social support (friends, family, significant other) [12], availability of social support [13], among others.

## Conclusion

The relationship between social support and the health of Aboriginal Australians is nuanced. The social support derived from extended families provides health benefits, but the high-density networks and lack of financial resources can create over-obligations and force harmful relationships. One main challenge to epidemiological research was the lack of psychological instruments validated to measure social support in this group. To the best of our knowledge, this is the first study to validate a measure of social support for an Indigenous population. The present study showed that SSS is a construct valid and reliable instrument to measure social support in Aboriginal Australians.

## Supporting information

S1 Fig GLLRMs of the SSS for Sample 1 (left) and Sample 2 (right).
(DOCX)

S2 Fig Item Map of the SSS in Sample 1 (left and center) and Sample 2 (right).
(DOCX)

S3 Fig Test Information Function for Sample 1 (left and center) and Sample 2 (right).
(DOCX)

S1 Table The SSS items. (DOCX)

S2 Table Item fit statistics for the RM and GLLRM of the Social Support Scale (SSS). (DOCX)

S3 Table Matrix of residual correlations of the Social Support Scale. (DOCX)

S4 Table Kelderman’s likelihood ratio tests for the GLLRM of the Social Support Scale. (DOCX)

S5 Table Targeting and reliability information of the SSS. (DOCX)

S6 Table Convergent and divergent validity of the SSS. (DOCX)
