# Supplementary material for: Psychometric properties of the Social Support Scale (SSS) in two Aboriginal samples
Source: PLoS One. 2023 Jan 3;18(1):e0279954. doi: 10.1371/journal.pone.0279954 (PMC9810148; doi:10.1371/journal.pone.0279954)
Supplement: S1 Fig — GLLRMs of the SSS for Sample 1 (left) and Sample 2 (right). (DOCX) [file pone.0279954.s001.docx]

**S1 Fig. GLLRMs of the SSS for Sample 1 (left) and Sample 2 (right).**

**
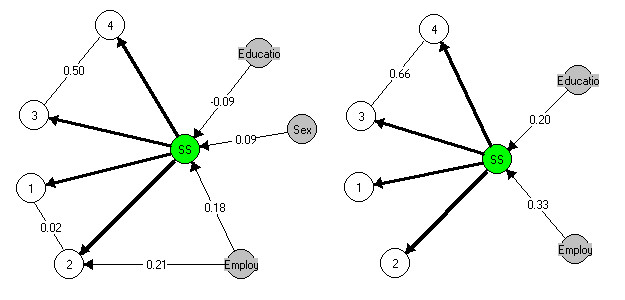
**

Note. The Markov graph nodes represent the item numbers, the exogenous variables, and the latent trait. Disconnected nodes indicate that variables are conditionally independent and partial $\gamma$ inform the magnitude of the LD and DIF.
