# Supplementary material for: Psychometric properties of the Social Support Scale (SSS) in two Aboriginal samples
Source: PLoS One. 2023 Jan 3;18(1):e0279954. doi: 10.1371/journal.pone.0279954 (PMC9810148; doi:10.1371/journal.pone.0279954)
Supplement: S2 Fig — Item Map of the SSS in Sample 1 (left and center) and Sample 2 (right). (DOCX) [file pone.0279954.s002.docx]

**S2 Fig. Item Map of the SSS in Sample 1 (left and center) and Sample 2 (right).**


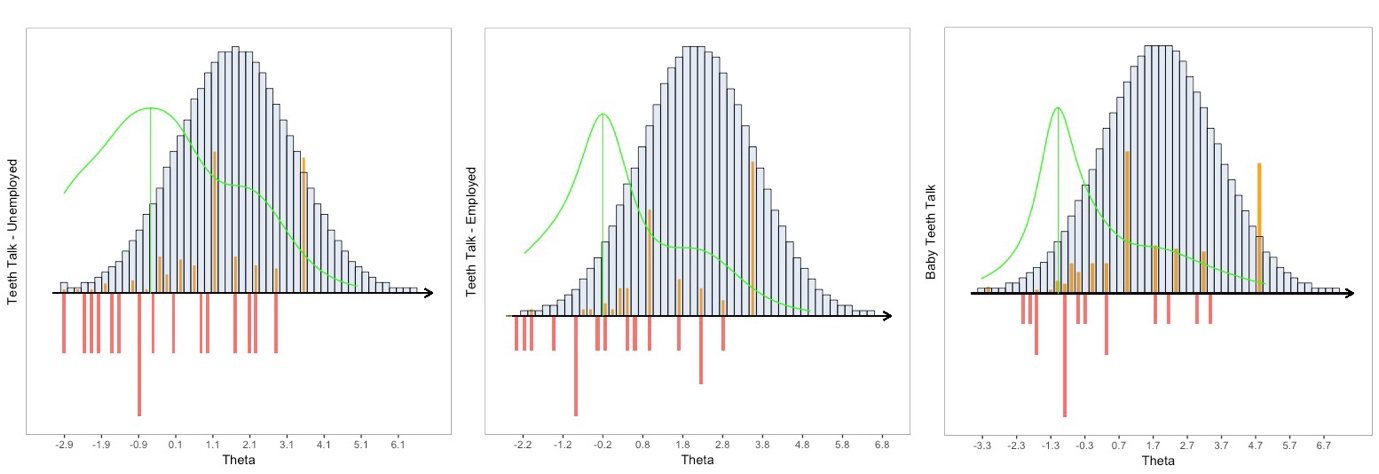


Note. The orange bars display the person parameters (WML estimates). The grey bars display the theoretical population distribution of social support under the assumption of normality. The red bars display the item thresholds and the green line is the information function.
