# Supplementary material for: Psychometric properties of the Social Support Scale (SSS) in two Aboriginal samples
Source: PLoS One. 2023 Jan 3;18(1):e0279954. doi: 10.1371/journal.pone.0279954 (PMC9810148; doi:10.1371/journal.pone.0279954)
Supplement: S3 Fig — Test Information Function for Sample 1 (left and center) and Sample 2 (right). (DOCX) [file pone.0279954.s003.docx]

**S3 Fig. Test Information Function for Sample 1 (left and center) and Sample 2 (right).**

**
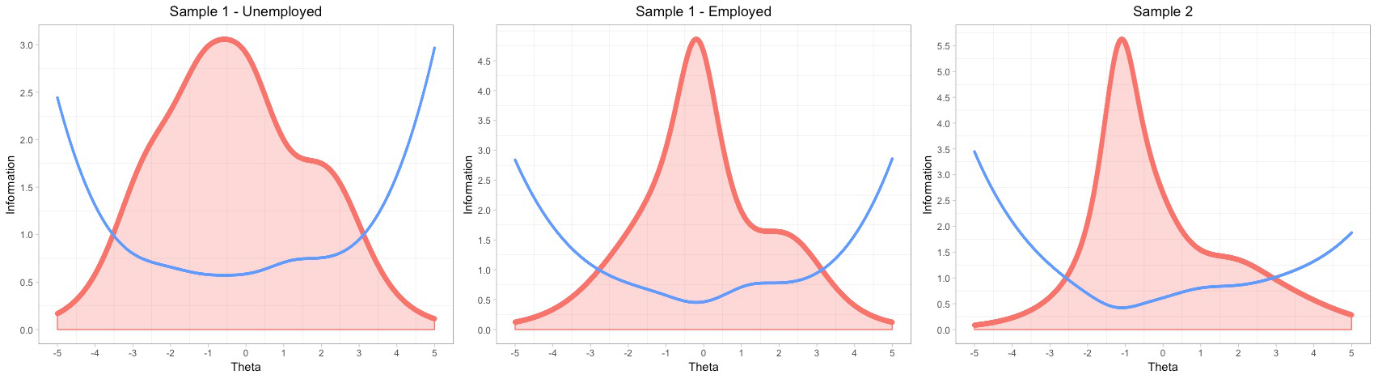
**

Note. The x-axis indicates the latent trait (“Social support”) and the y-axis indicates the information. The red line with shaded area represents the Test Information Function and the blue line represents the Standard Error of Measurement (SEM).
