# Supplementary material for: Psychometric properties of the Social Support Scale (SSS) in two Aboriginal samples
Source: PLoS One. 2023 Jan 3;18(1):e0279954. doi: 10.1371/journal.pone.0279954 (PMC9810148; doi:10.1371/journal.pone.0279954)
Supplement: S1 Table — (DOCX) [file pone.0279954.s004.docx]

**S1 Table. The SSS items.**

| Item number | Item content |
| --- | --- |
| 1 | There are people in my life who pay attention to my feelings and problems |
| 2 | There are people in my life who appreciate what I do |
| 3 | There are people in my life who I can get help from if I need it |
| 4 | There are people in my life who I can talk to about how to handle things |
