# Supplementary material for: Psychometric properties of the Social Support Scale (SSS) in two Aboriginal samples
Source: PLoS One. 2023 Jan 3;18(1):e0279954. doi: 10.1371/journal.pone.0279954 (PMC9810148; doi:10.1371/journal.pone.0279954)
Supplement: S2 Table — (DOCX) [file pone.0279954.s005.docx]

**S2 Table. Item fit statistics for the RM and GLLRM of the Social Support Scale (SSS).**

|  | Conditional Outfit | | | Conditional Infit | | |
| --- | --- | --- | --- | --- | --- | --- |
|  | Observed | SE | p-value | Observed | SE | p-value |
|  | Sample 1 | | | | | |
| RM |  |  |  |  |  |  |
| Item 1 | 1.350 | 0.103 | <0.001 | 1.319 | 0.100 | 0.001 |
| Item 2 | 0.794 | 0.110 | 0.060 | 0.815 | 0.106 | 0.080 |
| Item 3 | 0.912 | 0.104 | 0.400 | 0.920 | 0.103 | 0.438 |
| Item 4 | 0.954 | 0.107 | 0.665 | 0.978 | 0.104 | 0.823 |
| GLLRM |  |  |  |  |  |  |
| Item 1 | 1.254 | 0.133 | 0.056 | 1.234 | 0.124 | 0.058 |
| Item 2 | 0.730 | 0.124 | 0.029 | 0.773 | 0.130 | 0.079 |
| Item 3 | 0.965 | 0.120 | 0.714 | 0.952 | 0.121 | 0.691 |
| Item 4 | 0.988 | 0.121 | 0.921 | 1.046 | 0.113 | 0.585 |
|  | Sample 2 | | | | | |
| RM |  |  |  |  |  |  |
| Item 1 | 1.234 | 0.095 | 0.014 | 1.150 | 0.090 | 0.096 |
| Item 2 | 1.213 | 0.108 | 0.049 | 1.118 | 0.096 | 0.217 |
| Item 3 | 0.804 | 0.102 | 0.054 | 0.832 | 0.100 | 0.092 |
| Item 4 | 0.785 | 0.103 | 0.037 | 0.808 | 0.105 | 0.068 |
| GLLRM |  |  |  |  |  |  |
| Item 1 | 0.826 | 0.112 | 0.121 | 0.879 | 0.097 | 0.212 |
| Item 2 | 0.890 | 0.122 | 0.367 | 0.991 | 0.100 | 0.931 |
| Item 3 | 1.239 | 0.149 | 0.110 | 1.170 | 0.120 | 0.156 |
| Item 4 | 1.080 | 0.140 | 0.566 | 1.011 | 0.119 | 0.976 |

Note. The Conditional Outfit and Conditional Infit statistics have expected values equal to one under the Rasch model.
