# Supplementary material for: Psychometric properties of the Social Support Scale (SSS) in two Aboriginal samples
Source: PLoS One. 2023 Jan 3;18(1):e0279954. doi: 10.1371/journal.pone.0279954 (PMC9810148; doi:10.1371/journal.pone.0279954)
Supplement: S3 Table — (DOCX) [file pone.0279954.s006.docx]

**S3 Table. Matrix of residual correlations of the Social Support Scale.**

|  |  |  | Sample 1 |  |  |
| --- | --- | --- | --- | --- | --- |
|  |  | Item 1 | Item 2 | Item 3 | Item 4 |
| Item 1 |  | 1 |  |  |  |
| Item 2 | Obs | **-0.254** | 1 |  |  |
|  | Adj | **0.073** |  |  |  |
| Item 3 | Obs | -0.482 | -0.309 | 1 |  |
|  | Adj | -0.155 | 0.017 |  |  |
| Item 4 | Obs | -0.523 | -0.286 | **-0.105** | 1 |
|  | Adj | -0.196 | 0.041 | **0.221** |  |
|  |  |  | Sample 2 |  |  |
|  |  | Item 1 | Item 2 | Item 3 | Item 4 |
| Item 1 |  | 1 |  |  |  |
| Item 2 | Obs | **-0.185** | 1 |  |  |
|  | Adj | **0.140** |  |  |  |
| Item 3 | Obs | -0.413 | -0.484 | 1 |  |
|  | Adj | -0.088 | -0.159 |  |  |
| Item 4 | Obs | -0.453 | -0.407 | **-0.009** | 1 |
|  | Adj | -0.127 | -0.082 | **0.316** |  |

Note. The matrix includes: a) the observed correlation between standardized residuals after the influence of the latent trait (“Social Support”) was accounted by the Rasch model; and b) the mean-adjusted residual correlations, which are the difference between the observed residual correlations and the average residual correlation.
