# Supplementary material for: Psychometric properties of the Social Support Scale (SSS) in two Aboriginal samples
Source: PLoS One. 2023 Jan 3;18(1):e0279954. doi: 10.1371/journal.pone.0279954 (PMC9810148; doi:10.1371/journal.pone.0279954)
Supplement: S4 Table — (DOCX) [file pone.0279954.s007.docx]

**S4 Table. Kelderman’s likelihood ratio tests for the GLLRM of the Social Support Scale.**

|  | Conditional Likelihood Ratio test | | | Obs $\gamma$ |
| --- | --- | --- | --- | --- |
| **Sample 1**^a^ | Differential Item Functioning | | |  |
| Item 1 & Sex: | lr =   4.65 | df =   4 | p = 0.325 |  |
| Item 2 & Sex: | lr =  5.67 | df =   4 | p = 0.225 |  |
| Item 3 & Sex: | lr =   2.59 | df =   4 | p = 0.629 |  |
| Item 4 & Sex: | lr =   4.01 | df =   4 | p = 0.404 |  |
| Item 1 & Education: | lr =   6.86 | df =   4 | p = 0.143 |  |
| Item 2 & Education: | lr =  3.00 | df =   4 | p = 0.557 |  |
| Item 3 & Education: | lr =   2.29 | df =   4 | p = 0.582 |  |
| Item 4 & Education: | lr =   3.20 | df =   4 | p = 0.524 |  |
| Item 1 & Income: | lr =    7.81 | df =   4 | p = 0.989 |  |
| Item 3 & Income: | lr =    11.61 | df =   4 | p = 0.020 |  |
| Item 4 & Income: | lr = 4.93 | df =   4 | p = 0.294 |  |
|  |  |  |  |  |
|  | Local Dependence | | | |
| Item 1 & Item 3: | lr =   48.88 | df = 16 | p < 0.001 | 0.26  -0.45 |
| Item 1 & Item 4: | lr =   57.75 | df = 16 | p < 0.001 | -0.36  -0.54 |
| Item 2 & Item 3: | lr =   9.50 | df = 16 | p = 0.892 |  |
| Item 2 & Item 4: | lr =   39.97 | df = 16 | p = 0.001 | -0.03  0.15 |
| **Sample 2** | Differential Item Functioning | | |  |
| Item 1 & Education: | lr =   2.44 | df =   4 | p = 0.655 |  |
| Item 2 & Education: | lr =  12.71 | df =   4 | p = 0.013 |  |
| Item 3 & Education: | lr =   6.94 | df =   4 | p = 0.139 |  |
| Item 4 & Education: | lr =   2.39 | df =   4 | p = 0.665 |  |
| Item 1 & Income: | lr =    1.62 | df =   4 | p = 0.806 |  |
| Item 2 & Income: | lr =    6.11 | df =   4 | p = 0.191 |  |
| Item 3 & Income: | lr =    2.04 | df =   4 | p = 0.728 |  |
| Item 4 & Income: | lr = 3.29 | df =   4 | p = 0.511 |  |
|  |  |  |  |  |
|  | Local Dependence | | | |
| Item 1 & Item 2: | lr =   45.34 | df = 16 | p < 0.001 | 0.04  0.27 |
| Item 1 & Item 3: | lr =   52.69 | df = 16 | p = 0.104 |  |
| Item 1 & Item 4: | lr =   96.56 | df = 16 | p = 0.027 |  |
| Item 2 & Item 3: | lr =   91.18 | df = 16 | p = 0.029 |  |
| Item 2 & Item 4: | lr =   92.19 | df = 16 | p = 0.562 |  |

Note. After the Benjamini-Hochberg procedure, statistical significance was adjusted as *p* < 0.010 for Sample 1 and

*p* < 0.004 for Sample 2 for a 5% FDR.
