# Supplementary material for: Psychometric properties of the Social Support Scale (SSS) in two Aboriginal samples
Source: PLoS One. 2023 Jan 3;18(1):e0279954. doi: 10.1371/journal.pone.0279954 (PMC9810148; doi:10.1371/journal.pone.0279954)
Supplement: S5 Table — (DOCX) [file pone.0279954.s008.docx]

**S5 Table. Targeting and reliability information of the SSS.**

| Sample | |  | Score | | | Test Target  Index |  | Reliability | Probability of  Person  Separation |
| --- | --- | --- | --- | --- | --- | --- | --- | --- | --- |
|  | | n | Mean | SD | Target |  | Cronbach’s α |  |  |
| Unemployed (Sample 1) | | 237 | 12.38 | 3.18 | 7.47 | 0.57 | 0.89 | 0.83 | 0.79 |
| Employed (Sample 1) | | 80 | 13.16 | 2.89 | 7.64 | 0.34 | 0.86 | 0.80 | 0.75 |
| Sample 2 |  | 365 | 12.49 | 3.15 | 5.43 | 0.28 | 0.88 | 0.84 | 0.78 |

Note. The mean score is the average score for each subgroup. The target is the score which maximizes the information function. Reliability is the proportion of true score variance in relation to the total score variance. The probability of person separation is the probability that the scores of two random persons have the same rank order as their true person parameters.
