# Supplementary material for: Psychometric properties of the Social Support Scale (SSS) in two Aboriginal samples
Source: PLoS One. 2023 Jan 3;18(1):e0279954. doi: 10.1371/journal.pone.0279954 (PMC9810148; doi:10.1371/journal.pone.0279954)
Supplement: S6 Table — (DOCX) [file pone.0279954.s009.docx]

**S6 Table. Convergent and divergent validity of the SSS.**

|  | PS | 95% C.I | PC | 95% C.I |
| --- | --- | --- | --- | --- |
| Sample 1 | -0.04 | [-0.15, 0.07] | 0.12 | [0.01, 0.22] |
| Sample 2 | -0.21 | [-0.35, -0.06] | 0.28 | [0.13, 0.41] |

Note. The table displays the score correlations between SSS and complementary measures. PS = Perceived Stress; PC = Perceived Coping.
